# Black‐Phosphorus‐Incorporated Hydrogel as a Sprayable and Biodegradable Photothermal Platform for Postsurgical Treatment of Cancer

**DOI:** 10.1002/advs.201700848

**Published:** 2018-03-03

**Authors:** Jundong Shao, Changshun Ruan, Hanhan Xie, Zhibin Li, Huaiyu Wang, Paul K. Chu, Xue‐Feng Yu

**Affiliations:** ^1^ Institute of Biomedicine and Biotechnology Shenzhen Institutes of Advanced Technology Chinese Academy of Sciences Shenzhen 518055 P. R. China; ^2^ Department of Physics and Department of Materials Science & Engineering City University of Hong Kong Tat Chee Avenue Kowloon Hong Kong China

**Keywords:** 2D materials, black phosphorus, near‐infrared lasers, photothermal cancer therapy, thermosensitive hydrogels

## Abstract

Photothermal therapy (PTT) is a fledgling therapeutic strategy for cancer treatment with minimal invasiveness but clinical adoption has been stifled by concerns such as insufficient biodegradability of the PTT agents and lack of an efficient delivery system. Here, black phosphorus (BP) nanosheets are incorporated with a thermosensitive hydrogel [poly(d,l‐lactide)‐poly(ethylene glycol)‐poly(d,l‐lactide) (PDLLA‐PEG‐PDLLA: PLEL)] to produce a new PTT system for postoperative treatment of cancer. The BP@PLEL hydrogel exhibits excellent near infrared (NIR) photothermal performance and a rapid NIR‐induced sol–gel transition as well as good biodegradability and biocompatibility in vitro and in vivo. Based on these merits, an in vivo PTT postoperative treatment strategy is established. Under NIR irradiation, the sprayed BP@PLEL hydrogel enables rapid gelation forming a gelled membrane on wounds and offers high PTT efficacy to eliminate residual tumor tissues after tumor removal surgery. Furthermore, the good photothermal antibacterial performance prevents infection and this efficient and biodegradable PTT system is very promising in postoperative treatment of cancer.

## 1 Introduction

Surgery is the most common means to treat cancer but its success hinges on the removal of well‐defined and primary tumors located in nonvital tissue regions.^1^, ^2^, ^3^ Nevertheless, some forms of cancer are poorly defined with high metastasis and when they are detected from vital organs or tissues in the human body, surgery is quite challenging and may not be sufficiently effective due to the high recurrence rate after the treatment.^4^ To reduce the incidence of relapse, radiotherapy and chemotherapy are often implemented after surgery^5^ but serious complications and side effects may arise.^6^ In this respect, new treatment techniques besides radiotherapy and chemotherapy are desirable in clinical practice.

Photothermal therapy (PTT) is a novel cancer therapy in which cancerous tissues are ablated based on the thermal effect of exogenous nanoagents under near‐infrared (NIR) irradiation.^7^ Compared to other conventional therapeutic techniques, PTT boasts many merits such as the simplicity, minimal invasiveness, low incidence of complications, as well as the high spatial and temporal precision of NIR light.^8^, ^9^, ^10^, ^11^ Various types of nanoagents have been explored to attain better PTT performance^12^, ^13^, ^14^, ^15^, ^16^, ^17^, ^18^, ^19^, ^20^, ^21^, ^22^, ^23^, ^24^ and in the treatment, intravenous injection of the nanoagents (to mice, for example) is followed by high‐power NIR laser irradiation for ablation.^25^, ^26^, ^27^, ^28^, ^29^ Despite recent progress, clinical adoption of PTT still faces several hurdles. First of all, most of the PTT nanoagents reported so far lack biodegradability and these residual nanoagents may accumulate in vital organs such as the liver, spleen, and kidney causing deleterious effects.^30^, ^31^ Second, malignant tumors usually are several centimeters or larger in size which is far beyond the penetration depth of NIR light (less than 1 cm)^32^ and so not the entire tumor can be irradiated. Therefore, new PTT techniques that can be combined with surgical treatment are imperative so that it can become more acceptable clinically.

In this study, a sprayable PTT system is designed by incorporating black phosphorus (BP) nanosheets with a thermosensitive hydrogel [Poly(d,l‐lactide)‐poly(ethylene glycol)‐poly(d,l‐lactide) (PDLLA‐PEG‐PDLLA: PLEL)] for postoperative photothermal treatment of cancer. BP nanosheets are a type of 2D nanomaterials with unique properties such as a tunable band gap, high NIR absorption, and high photothermal conversion efficiency.^33^, ^34^, ^35^, ^36^, ^37^, ^38^, ^39^, ^40^ As an inorganic nanoagent, BP nanosheets are attractive due to the inherent biocompatibility since phosphorus is a vital element, especially in bones, making up about 1% of the human body weight.^41^, ^42^, ^43^ Moreover, BP nanosheets degrade naturally in the physiological environment forming harmless PO_4_^3−^ as the final degradation product.^44^, ^45^, ^46^ Here, by combining BP with PLEL which is a biodegradable and biocompatible thermosensitive hydrogel,^47^, ^48^ the BP@PLEL is photothermally responsive to NIR irradiation and the cross‐linked gel structure is formed as the temperature is increased. In vitro and in vivo experiments are performed to investigate the biodegradability and biocompatibility of the BP@PLEL hydrogel and a PTT postoperative treatment strategy is described to prevent the recurrence of cancer.

## 2 Results and Discussion

### 2.1 Synthesis and Characterization of BP Nanosheets

**Figure 1:**
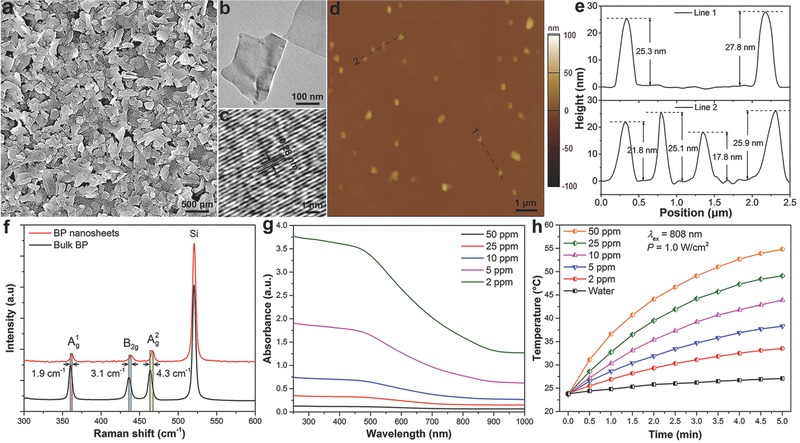
Characterization of BP nanosheets: a) SEM, b) TEM, c) high‐resolution TEM, and d) AFM images of the BP nanosheets; e) height profiles along the dashed lines in (d); f) Raman scattering spectra acquired from the bulk BP and BP nanosheets; g) absorption spectra of the BP nanosheets with different concentrations (2, 5, 10, 25, and 50 ppm); h) photothermal heating curves of the BP nanosheets with different concentrations (0, 2, 5, 10, 25, and 50 ppm) irradiated with an NIR laser (808 nm, 1.0 W cm^−2^) for 5 min.

The BP nanosheets are prepared by a modified liquid exfoliation technique based on the method reported by our group previously^49^, ^50^ and characterized by scanning electron microscopy (SEM), transmission electron microscopy (TEM), atomic force microscopy (AFM), and Raman scattering spectroscopy. As shown in **Figure** **1**a–e, the BP nanosheets have a uniform 2D sheet‐like morphology with an average lateral size of 288.3 ± 122.4 nm and thickness of 23.4 ± 8.2 nm according to the statistical analysis of 200 BP nanosheets. The high‐resolution TEM image shows lattice fringes with a *d*‐spacing of 0.28 nm (Figure 1c) matching those of the monolayered BP structure.^51^ Raman scattering (Figure 1f) reveals three prominent peaks at 362.3, 438.5 and 466.9 cm^−1^ associated with the out‐of‐plane phonon mode (A^1^_g_) and two in‐plane modes (B_2g_ and A^2^_g_) of BP,^52^ respectively. Compared to bulk BP, the A^1^_g_, B_2g_, and A^2^_g_ modes of the BP nanosheets redshift by about 1.9, 3.1, and 4.3 cm^−1^, respectively, further demonstrating successful preparation of the BP nanosheets.

The optical properties of the BP nanosheets dispersed in an aqueous solution are determined. As shown in Figure 1g, the BP nanosheets exhibit a typical broad absorption band spanning the ultraviolet and NIR regions similar to other 2D layered materials.^53^, ^54^ The extinction coefficient of the BP nanosheets is 31.4 Lg^−1^ cm^−1^ according to Beer's law,^55^ which is larger than those of most reported photothermal nanomaterials such as Au nanorods, GO nanosheets, WS_2_ nanosheets, and BP quantum dots.^56^, ^57^, ^58^, ^59^

To evaluate the NIR photothermal performance, different concentrations of BP nanosheets (0, 2, 5, 10, 25, and 50 ppm) are dispersed in aqueous solutions and exposed to an NIR laser (808 nm, 1.0 W cm^−2^). The solution temperature is monitored as a function of irradiation time (Figure 1h). At a low concentration (50 ppm), the solution temperature increases by 31.4 °C after irradiation for only 5 min. In contrast, the temperature of water increases by only 2.9 °C, indicating that the BP nanosheets can rapidly and efficiently convert NIR light into thermal energy.

### 2.2 Preparation and Thermogellability of the BP‐Incorporated Hydrogel

**Figure 2:**
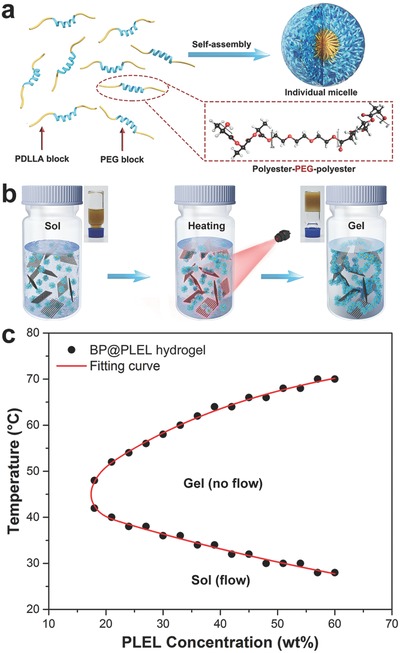
Thermogellability of the BP@PLEL hydrogel: a) schematic diagram of self‐assembly of PLEL into micelles; b) schematic representation of the thermogelation process of the BP@PLEL hydrogel induced by NIR irradiation; c) sol–gel phase transition diagram of the BP@PLEL hydrogel.

Temperature‐sensitive polymeric hydrogels have been extensively investigated for sustained drug delivery, cell encapsulation, tissue regeneration, and postoperative prevention.^60^, ^61^, ^62^ Typically, the aqueous polymer solutions at room temperature or below can spontaneously turn into nonflowing gels in response to the physiological temperature. Here, PLEL, a typical biodegradable and biocompatible temperature‐sensitive hydrogel,^47^, ^48^ is selected as the agent to load the BP nanosheets. As shown in **Figure** **2**a, the polymeric chains of PLEL can self‐assemble into core–shell like micelles in the aqueous solution. The hydrophobic PDLLA constitutes the core and the hydrated PEG forms the hydrophilic shell. Figure 2b shows the schematic illustration of the interactions between polymer micelles and BP nanosheets, which serves as the bridges between the polymer micelles due to their large surface area. Furthermore, the BP@PLEL is photothermally sensitive to NIR irradiation and the physically cross‐linked gel structure forms as the temperature is increased. The SEM and TEM images of the BP@PLEL hydrogel are displayed in Figures S1 and S2 (Supporting Information), respectively, revealing the uniform distribution of BP nanosheets in PLEL hydrogel.

The temperature dependent solution–gel (sol–gel) phase transition is investigated by the test‐tube‐inversion method. Different amounts of PLEL are dissolved in water at ambient temperature followed by addition of 50 ppm BP nanosheets and subsequent sol–gel transition as the temperature goes up. The sol–gel phase transition diagram of the BP@PLEL hydrogel in Figure 2c shows that as PLEL concentration is increased, gelation takes place at a lower temperature (lower critical gelation temperature) and precipitation occurs at a higher temperature (upper critical gelation temperature). Moreover, there were no significant differences between the BP@PLEL hydrogel and pure PLEL hydrogel in the phase diagrams (Figure S3, Supporting Information) and dynamic rheological measurements (Figure S4, Supporting Information), indicating that the introduction of BP nanosheets has an insignificant effect to the gelation of PLEL hydrogel. According to our experiments, the BP@PLEL with a concentration of 30 wt% shows a gelation temperature of about 37 °C and thus chosen for the following experiments.

### 2.3 Photothermal‐Induced Gelation of the BP@PLEL Hydrogel

**Figure 3:**
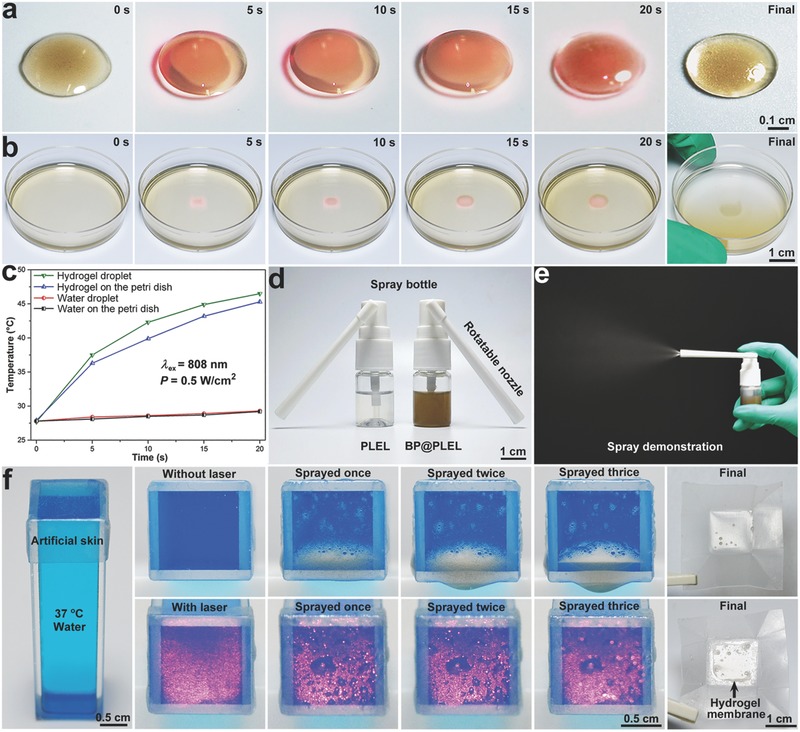
Photothermal‐induced gelation and sprayability of the BP@PLEL hydrogel: a) photographs of a single BP@PLEL hydrogel droplet (100 µL) and b) photographs of 2 mL of the BP@PLEL hydrogel on a petri dish irradiated with the 808 nm laser (0.5 W cm^−2^) for different periods of time; c) corresponding photothermal heating curves of water and hydrogel in (a) and (b); d) photograph of the PLEL and BP@PLEL hydrogel separately loaded into a 5 mL medical throat spray bottle; e) spraying of the BP@PLEL hydrogel; f) photographs showing the of BP@PLEL hydrogel sprayed on a piece of 37 °C artificial skin with (down) and without (up) 808 nm laser irradiation.

Since the 808 nm semiconductor laser is cheap and commonly used in PTT studies, an 808 nm fiber‐coupled continuous semiconductor diode laser has been used in the experiments. **Figure** **3**a,b illustrates the gelation performance of the BP@PLEL hydrogel droplet (100 µL) and BP@PLEL hydrogel on the petri dish (2 mL) irradiated with the 808 nm laser (0.5 W cm^−2^) and the temperature rise is monitored as a function of illumination (Figure 3c). During NIR irradiation, the BP@PLEL hydrogel droplet undergoes the sol–gel transition within a few seconds and gelation is completed after only 20 s (Figure 3a) as the temperature rises from 27.8 to 46.5 °C (Figure 3c). When the NIR laser is adjusted to a spot size of 0.5 cm^2^ and focused onto the central part of the BP@PLEL hydrogel (Figure 3b), only a small portion near the center can be gelled after 20 s with the local temperature going up from 27.9 to 45.5 °C. The results demonstrate that the sol–gel transition of BP@PLEL hydrogel can be precisely controlled by NIR light by simply tailoring the irradiation time and location site.

Figure 3d shows the PLEL and BP@PLEL hydrogel separately loaded in a 5 mL medical throat spraying bottle and the spraying process of the BP@PLEL hydrogel is presented in Figure 3e. The BP@PLEL hydrogel shows good fluidity at room temperature thus boding well for clinical application at room temperature. To simulate actual clinical conditions, a piece of commercialized artificial skin (polylactic acid) covers the cuvette filled with 37 °C water and the BP@PLEL hydrogel is vertically sprayed onto the surface of artificial skin with or without NIR laser irradiation. The sprayed hydrogel without NIR irradiation gradually slides off the surface but conversely, the sprayed hydrogel after NIR irradiation forms a gelled membrane on the artificial skin rapidly, demonstrating that the BP@PLEL hydrogel is very sensitive to NIR irradiation undergoing rapid in situ gelation locally. Such hydrogel formulation is quite suitable for the therapy of wound surface by perfect matching, whereas a flowable solution will cause uneven distribution.

### 2.4 Degradation Performance of the BP@PLEL Hydrogel

**Figure 4:**
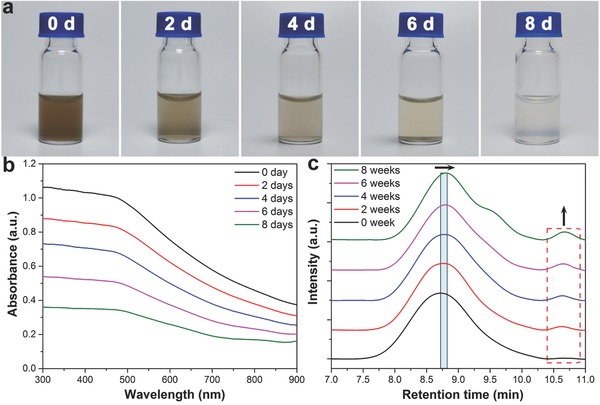
Degradation performance of the BP@PLEL hydrogel: a) photographs and b) absorption spectra of the BP@PLEL hydrogel stored for different periods of time (0, 2, 4, 6, and 8 d); c) GPC profiles of the copolymers in the BP@PLEL hydrogel after storage for 0, 2, 4, 6, and 8 weeks. The right arrow refers to decreasing peak molecular weight and the dashed circle refers to the emergence of degradation products with small molecular weights.

To evaluate degradation of BP in the PLEL hydrogel, the BP@PLEL hydrogel is placed in a horizontal shaker at 37 °C for 8 d and the optical properties are examined at predetermined time intervals of 0, 2, 4, 6, and 8 d. As shown in **Figure** **4**a, the color of the BP@PLEL hydrogel fades gradually and is almost transparent and colorless after 8 d indicative of complete degradation. The absorption spectra in Figure 4b show that the absorption intensity decreases with degradation time further confirming rapid degradation of BP in the PLEL hydrogel. Degradation of BP is caused by the irreversible reaction with oxygen and water, forming oxidized phosphorus species (P→P*_x_*O*_y_*) followed by the subsequent reaction of P*_x_*O*_y_* to the final products, that is, (PO_4_^3−^) ions.^63^ Although the BP nanosheets maintain the stability in the hydrogel for only few days, but from another perspective, they retain the outstanding degradability after incorporation with the hydrogel, which is very desirable for the bioapplications. In fact, the BP nanosheets can be stored in a vacuum environment for a much longer time and employed to prepare the BP@PLEL hydrogel by simply mixing them with the PLEL hydrogel before using.

The long‐term degradation behavior of the BP@PLEL hydrogel is evaluated under the mimetic physiological conditions of 37 °C and pH of 7.4. At predetermined time intervals (0, 2, 4, 6, and 8 weeks), the remaining hydrogels are collected and measured by gel permeation chromatography (GPC). As shown in Figure 4c, the retention time of the maximum peak rightshifts slightly and the chromatograms broaden gradually as a function of degradation time. Multimodes emerge during degradation (see the red dashed rectangle in Figure 4c) possibly due to degradation products with small molecular weights. According to previous studies,^47^ degradation of PLEL hydrogels is proceeded by steady hydrolysis of the ester linkage into segments (reduced molecular weight), oligomers and monomers, and finally dissociation to carbon dioxide and water. The BP@PLEL hydrogel with reasonable biodegradability not only provides excellent wound‐healing performance, but also enables clearance of the BP@PLEL hydrogel after fulfilling the therapeutic functions.

### 2.5 Biocompatibility of the BP@PLEL Hydrogel

**Figure 5:**
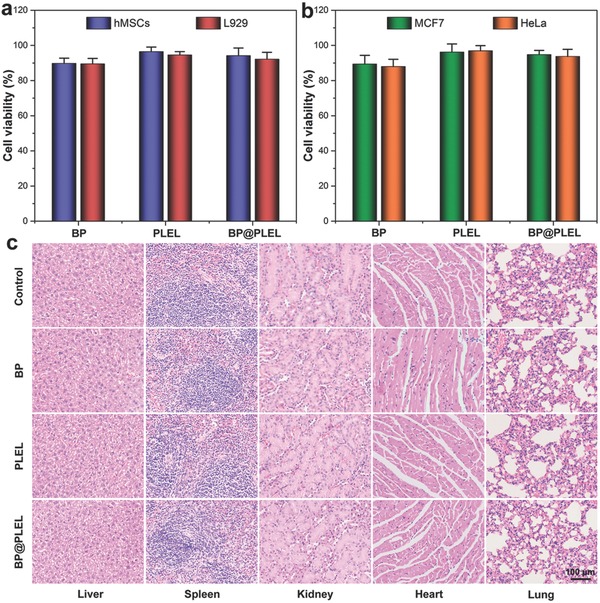
In vitro and in vivo biocompatibility of the BP nanosheets, PLEL hydrogel, and BP@PLEL hydrogel: relative viability of a) normal cells and b) tumor cells incubated with different samples at 37 °C for 48 h; c) histological data obtained from the liver, spleen, kidney, heart, and lung of the mice injected with the BP nanosheets, PLEL hydrogel, and BP@PLEL hydrogel at 20 d postinjection.

To assess possible cytotoxicity, human mesenchymal stem cells (hMSCs), mouse fibroblast cells (L929), human breast cancer cells (MCF7), and human cervical cancer cells (HeLa) are separately incubated with the BP nanosheets, PLEL hydrogel, and BP@PLEL hydrogel at 37 °C for 48 h and studied using the cell counting kit‐8 (CCK‐8) assay. **Figure** **5**a,b does not disclose significant cytotoxicity from all types of cells corroborating the good biocompatibility of the BP@PLEL hydrogel in vitro.

The potential toxicity of the BP nanosheets, PLEL hydrogel, as well as BP@PLEL hydrogel is also systematically investigated in vivo. Twenty healthy female Balb/c mice (6 weeks old) are randomly divided into four groups: (1) control group without any treatment, (2) BP nanosheets, (3) PLEL hydrogel, and (4) BP@PLEL hydrogel subcutaneously injected into the rear parts of mice. At 20 d postinjection, the major organs including the liver, spleen, kidney, heart, and lung are collected from the different experimental groups and sliced for hematoxylin and eosin staining. Figure 5c demonstrates no apparent histological abnormality or lesion and the BP@PLEL hydrogel has insignificant toxicity in vivo.

### 2.6 In Vivo PTT for Postoperative Treatment of Cancer

**Figure 6:**
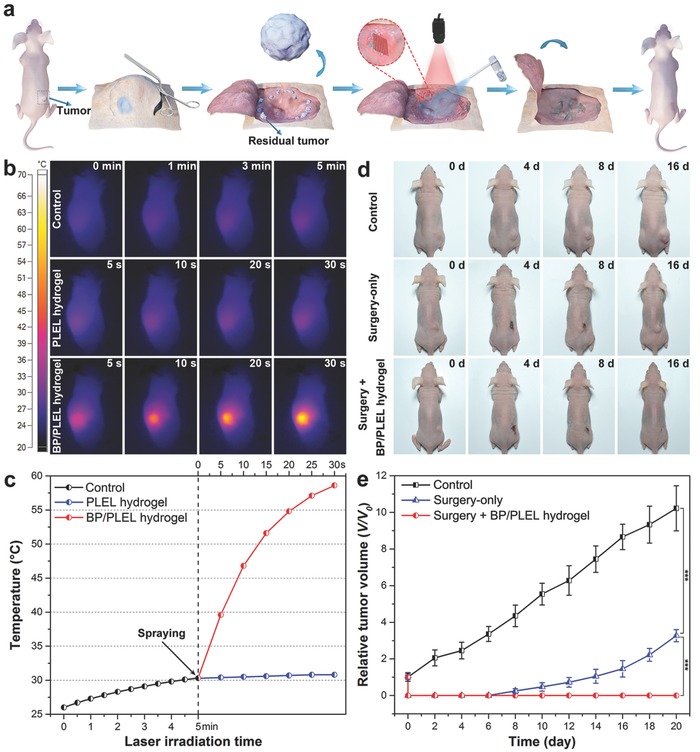
In vivo PTT for postoperative treatment of cancer: a) schematic representation of the surgical treatment and postoperative PTT of the tumor; b) infrared thermographic maps and c) time‐dependent temperature increase of the tumor‐bearing nude mice sprayed with the PLEL and BP@PLEL hydrogel followed by illumination with the 808 nm laser (0.5 W cm^−2^); d) typical photographs and e) corresponding growth curves of the tumor‐bearing mice without any treatment, surgical treatment only, and surgical treatment and postoperative PTT (***P < 0.001).

To evaluate the potential of BP@PLEL hydrogel in postoperative treatment of cancer by PTT, tumor models are established in situ by subcutaneous injection of HeLa cells into the right rear flank of the Balb/c nude mice. When the tumor volume reaches ≈100 mm^3^, 15 tumor‐bearing mice are randomly divided into three groups: (1) control group without any treatment, (2) tumor‐bearing mice undergoing tumor removal surgery only, and (3) tumor‐bearing mice undergoing tumor removal surgery and postoperative PTT treatment. As shown in **Figure** **6**a, a skin incision is made at the edge of the tumor site and the main tumor body is surgically excised. Afterward, the entire wound region is irradiated with the 808 nm laser (0.5 W cm^−2^), sprayed with the BP@PLEL hydrogel, and sutured after the PTT treatment. The infrared thermographic maps are recorded by an infrared thermal imaging camera and time‐dependent changes in the tumor temperature are monitored simultaneously (Figure 6b,c). By NIR irradiation of the tumor‐bearing sites for different time periods, the temperature increase observed from the control group is only 4.3 °C after 5 min and that of PLEL‐sprayed group is only 0.8 °C after 30 s. In contrast, the temperature of the BP@PLEL group rises rapidly to 39.4 °C during the first 5 s of NIR irradiation that is beyond the temperature for BP@PLEL hydrogel gelation. The temperature reaches 58.2 °C within 30 s, which is high enough for photothermal ablation of cancer cells.^64^

The tumor‐bearing mice in the different experimental groups are monitored for signs of distress daily and the tumor volume is measured every 2 d. Neither death nor obvious sign of toxic side effects such as abnormal body weight, drinking, or eating are observed during the posttherapy period. As shown in Figure 6d,e, the tumor‐bearing mice after surgery and PTT treatment are completely cured without recurrence within 16 d and can survive for over two months without a single death. In contrast, the tumor‐bearing mice undergoing tumor removal surgery exhibit an extremely high local recurrence rate (80%) after 8 d and have a life span of about 35 d. The tumor bearing mice without any treatment only have a life span of 18–24 d and the average tumor volume reaches 1000 mm^3^ in 20 d. These results clearly demonstrate the excellent PTT efficacy of the BP@PLEL hydrogel.

### 2.7 Photothermal Antibacterial Performance of BP@PLEL Hydrogel

**Figure 7:**
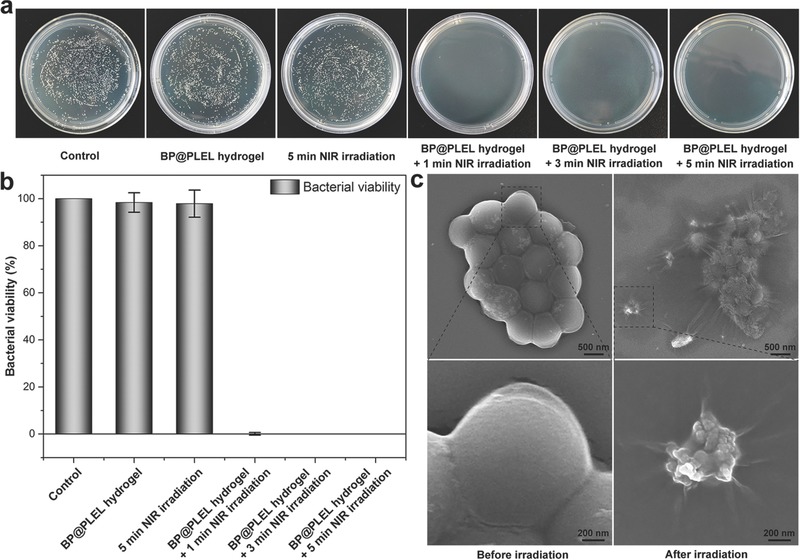
Photothermal antibacterial performance: a) bacterial colonies and b) corresponding bacterial viability of *S. aureus* of the blank control group, BP@PLEL hydrogel group, 5 min NIR irradiation group, BP@PLEL hydrogel + 1 min NIR irradiation group, BP@PLEL hydrogel + 3 min NIR irradiation group, and BP@PLEL hydrogel + 5 min NIR irradiation group; c) typical morphology of the bacteria incubated with the BP@PLEL hydrogel before and after laser irradiation.

Wound infection caused by bacteria is one of the most prevalent complications after surgery.^65^ As an efficient approach for sterilization, PTT has also been extensively investigated in antibacterial therapy.^66^, ^67^, ^68^ Here, the photothermal antibacterial properties of the BP@PLEL hydrogel is studied by the colony formation assay and 1, 3, or 5 min NIR irradiation. As shown in **Figure** **7**a,b, the bacteria colonies on the BP@PLEL hydrogel + NIR irradiation groups are much less than those on the blank control group, BP@PLEL hydrogel group, and 5 min NIR irradiation group. The BP@PLEL hydrogel or NIR laser irradiation alone is harmless to *S. Aureus*, but the photothermal performance of BP@PLEL hydrogel + NIR laser irradiation is sufficient to sterilize more than 99.5% of the bacteria. Figure 7c depicts the typical morphology of the bacteria incubated with the BP@PLEL hydrogel before and after irradiation. The bacteria incubated with the BP@PLEL hydrogel remain intact and smooth before NIR irradiation but after NIR irradiation, the bacteria become seriously wrinkled and flattened and the cellular membranes break down severely. The results demonstrate that the BP@PLEL hydrogel is also a rapid (about 1 min) and effective (>99.5% killing efficiency) photothermal antibacterial agent in the prevention of postoperative wound infection after cancer treatment.

## 3 Conclusions

The BP@PLEL hydrogel is designed and adopted in a sprayable and biodegradable PTT system for postsurgical treatment of cancer. The BP@PLEL hydrogel delivers excellent NIR PTT performance, facilitates rapid sol–gel transition under NIR irradiation, and has excellent biodegradability and biocompatibility as verified by in vitro and in vivo experiments. The sprayed BP@PLEL hydrogel under NIR irradiation enables rapid gelation forming a gelled membrane on wound and has high PTT efficacy to eliminate residual tumor tissues. Compared to other previously reported PTT agents, the BP@PLEL hydrogel is especially attractive due to the biodegradability and biocompatibility. Both BP nanosheets and PLEL are biodegradable and all the degradation products are safe small molecules including phosphate, phosphonate, carbon dioxide, and water, which can be excreted harmlessly from the body after the therapy. Based on the BP@PLEL hydrogel, a new PTT treatment strategy is proposed and demonstrated by spraying only a small amount of the hydrogel onto the tumor site followed by low‐dose NIR laser irradiation (0.5 W cm^−2^). Compared to radiotherapy and chemotherapy, this postoperative treatment is simpler and causes less side effects. Furthermore, this technique not only eliminates residual tumor tissues, but also can prevent wound infection. It thus has immense clinical potential in the treatment of cancer.

## 4 Experimental Section

*Materials*: The BP crystals were purchased from a commercial supplier (Smart‐Elements) and stored in a dark Ar glove box and *N*‐methyl‐2‐pyrrolidone (NMP, 99.5%, anhydrous) was obtained from Aladdin Reagents. The triblock PDLLA‐PEG‐PDLLA copolymers with a molecular weight of 4500 Da were purchased from PolySciTech (Indiana, USA). All other chemicals used in this study were analytical reagent grade and used without further purification.

*Synthesis of the BP Nanosheets*: The BP nanosheets were prepared by a modified liquid exfoliation technique. Briefly, the BP crystals were dispersed in NMP at an initial concentration of 1 mg mL^−1^ and ground to fine powders. The dispersion was sonicated in an ice bath for 10 h using a power of 300 W and centrifuged at 4000 rpm for 10 min. The supernatant containing the BP nanosheets was decanted gently and centrifuged for another 10 min at 7000 rpm. The precipitate was collected and resuspended for further experiments.

*Characterization of the BP Nanosheets*: The SEM images were obtained on the field‐emission SEM (NOVA NANOSEM430, FEI, The Netherlands) at 3–5 kV and TEM was conducted on the JEOL JEM‐2010 TEM at 200 kV. AFM was performed on an MFP‐3D‐S AFM (Asylum Research, USA) using the tapping mode in air. The Raman scattering spectra were obtained on the Horiba Jobin‐Yvon LabRam HR‐VIS high‐resolution confocal Raman microscope equipped with the 633 nm laser as the excitation source. The UV–vis–NIR absorption spectra were acquired on a Lambda25 spectrophotometer (PerkinElmer) with QS‐grade quartz cuvettes at room temperature. The BP concentration was determined by inductively coupled plasma atomic‐emission spectroscopy (7000DV, PerkinElmer). To study the photothermal effects of BP nanosheets, the samples with different concentrations were maintained in a 1 cm path length quartz cuvette and irradiated with a fiber‐coupled continuous semiconductor diode laser (808 nm, KS‐810F‐8000, Kai Site Electronic Technology Co., Ltd. Shaanxi, China) at a power density of 1.0 W cm^−2^ for 5 min. The laser spot was adjusted to fully cover the entire surface of the sample and the temperature change was recorded by an infrared thermal imaging camera (Fluke TiS75, USA).

*Preparation of the BP@PLEL Hydrogel*: The molecular weight of the PLEL hydrogel was determined by GPC with multiangle laser light scattering (Malvern Viscotek 270max). 10 mL of the 50 ppm BP nanosheets in NMP were centrifuged for 10 min at 7000 rpm, and the precipitate was washed thrice with deionized water by centrifugation at 7000 rpm for 10 min to remove residual NMP. Afterward, the BP nanosheets were redispersed in 10 mL of the 30% (w/v) PLEL solution by sonication to obtain the BP@PLEL hydrogel.

*Phase Diagram of the BP@PLEL Hydrogel*: The sol–gel phase transition temperature of the BP@PLEL aqueous solution was determined by the test‐tube‐inversion method. The aqueous solution was maintained in a 2 mL centrifuge tube (inner diameter of 10 mm) with a temperature increment of 1 °C per step from 20 °C to the temperature when precipitation occurred. The sol–gel phase transition was defined when no significant flow was observed visually by inverting the vials.

*Photothermal Gelation of the BP@PLEL Hydrogel*: 100 µL of the BP@PLEL hydrogel droplet on the slide and 2 mL of the hydrogel in a 60 mm petri dish were irradiated with the 808 nm laser (0.5 W cm^−2^, spot size: 0.5 cm^2^). The temperature of samples was monitored as a function of time by the Fluke TiS75 infrared thermal imaging camera and the corresponding photographs were captured by a Nikon digital camera. To simulate clinical conditions, the BP@PLEL hydrogel was sprayed onto the surface of vertically placed artificial skin (Lando Biomaterials Co., Ltd, Shenzhen, China) covered on a cuvette filled with 37 °C water followed by illumination with the 808 nm laser (0.5 W cm^−2^) for gelation. A water‐soluble blue dye was dissolved in the 37 °C water to make performances more perceptible.

*In Vitro Degradation of the BP@PLEL Hydrogel*: To investigate the degradation characteristics, 2 mL of the BP@PLEL hydrogel placed in closed sample vials were maintained in a horizontal shaker at 37 °C for 8 d and the optical properties were determined at predetermined time intervals of 0, 2, 4, 6, and 8 d. The remaining gels were taken out at predetermined times and analyzed by GPC.

*In Vitro Cytotoxicity Assays*: The hMSCs, L929, MCF7, and HeLa cells were obtained from the American Type Culture Collection. The cells were seeded on a 96‐well plate at a density of 1 × 10^4^ cells per well and maintained with 150 µL of the H‐DMEM medium (Gibco BRL) supplemented with 10% (v/v) fetal bovine serum in a humidified atmosphere of 37 °C with 5% CO_2_. After culturing overnight, the medium was replaced with 150 µL of the H‐DMEM medium containing the BP nanosheets (50 ppm), PLEL hydrogel (30% (w/v)), and BP@PLEL hydrogel (50 ppm BP and 30% (w/v) hydrogel), respectively. Five multiple wells were used for each sample. After incubation for 48 h, the relative cell viability of the different experimental groups was determined by a CCK‐8 assay (Donjindo) according to the manufacturer's instruction.

*In Vivo Toxicity Assays*: Twenty healthy female Balb/c mice (6 weeks old) were purchased from Slac Laboratory Animal Co., Ltd. (Hunan, China) and all the in vivo experiments followed the protocols approved by the Animal Care and Use Committee of the Shenzhen Institutes of Advanced Technology, Chinese Academy of Sciences. 200 µL aliquots of the 50 ppm BP nanosheets, 30% (w/v) PLEL hydrogel and BP@PLEL hydrogel with 50 ppm BP, and 30% (w/v) PLEL hydrogel were subcutaneously injected into the rear parts of the mice, respectively. The different experimental groups were monitored and compared with the control group of mice injected with the phosphate‐buffered saline (PBS) solution. At 20 d postinjection, the mice were sacrificed and the major organs (heart, liver, spleen, lung, and kidney) were harvested, fixed in 10% neutral buffered formalin, processed routinely into paraffin, sectioned at 8 mm, stained with hematoxylin and eosin, and examined by digital microscopy.

*In Vivo PTT of Cancer*: 1 × 10^7^ HeLa cancer cells in 100 µL of PBS were subcutaneously injected into the right rear flank of each Balb/c nude mice (6 weeks old) to establish the tumor‐bearing mice model. When the tumor volume reached ≈100 mm^3^, 15 mice were randomly divided into three groups: (1) control group without any treatment, (2) mice undergoing tumor removal surgery only, and (3) mice undergoing tumor removal surgery and postoperative PTT treatment. For the mice undergoing tumor removal surgery, a skin incision was made at the edge of the tumor after the mice were anesthetized, and the main tumor body was surgically excised along the tumor border with the normal tissues. Afterward, the wound was routinely sutured and coated with antibiotic ointment to avoid postoperative infection. For the mice undergoing additional postoperative PTT treatment, the entire wound region was irradiated with the 808 nm laser (0.5 W cm^−2^), sprayed by the BP@PLEL hydrogel (containing the BP nanosheets (50 ppm), PLEL hydrogel (30% (w/v)) and then sutured after the PTT treatment. The tumor size of the mice was measured by a caliper every 2 d and the relative tumor volume was determined by *V/V*_0_ where *V*_0_ was the initial tumor volume before NIR irradiation.

*Photothermal Antibacterial Performance of BP@PLEL Hydrogel*: The Gram‐positive *S. aureus* bacteria were obtained from China Center of Industrial Culture Collection and grown in Luria–Bertani (LB) media overnight at 37 °C with 150 rpm rotation. When the concentration of bacteria reached 10^8^ CFU mL^−1^ (OD_600_ = 0.1), the bacterial suspensions were centrifuged at 6000 rpm, washed twice with 0.01 m PBS solution, and resuspended in the LB media containing the BP@PLEL hydrogel. After incubation for 2 h, the mixtures were irradiated with an 808 nm laser at a power of 0.5 W cm^−2^ for 0, 1, 3, or 5 min. The resulting bacterial suspensions were serially diluted and 100 µL of each sample were added to LB agar plates and cultured at 37 °C for 12 h. The bacteria viability was determined by the standard plate‐based counting method. The morphological changes of the *S. aureus* before and after laser irradiation were monitored by SEM after sample fixing and dehydration.

*Statistical Analysis*: The experimental results were presented as means ± standard deviation and the significance of difference was analyzed by variance statistics. In all the statistical evaluations, *P* < 0.05 was considered significant, *P* < 0.01 was considered highly significant, and *P* < 0.001 was considered very highly significant.

## Conflict of Interest

The authors declare no conflict of interest.

## Supporting information

Supplementary

## References

[1] S. Keereweer , P. B. Van Driel , T. J. Snoeks , J. D. Kerrebijn , R. J. B. de Jong , A. L. Vahrmeijer , H. J. Sterenborg , C. W. Löwik , Clin. Cancer Res. 2013, 14, 3745. 10.1158/1078-0432.CCR-12-3598 23674494

[2] C. E. DeSantis , C. C. Lin , A. B. Mariotto , R. L. Siegel , K. D. Stein , J. L. Kramer , R. Alteri , A. S. Robbins , A. Jemal , Ca‐Cancer J. Clin. 2014, 64, 252. 24890451 10.3322/caac.21235

[3] K. D. Miller , R. L. Siegel , C. C. Lin , A. B. Mariotto , J. L. Kramer , J. H. Rowland , K. D. Stein , R. Alteri , A. Jemal , Ca‐Cancer J. Clin. 2016, 66, 271. 27253694 10.3322/caac.21349

[4] M. Reig , Z. Mariño , C. Perelló , M. Iñarrairaegui , A. Ribeiro , S. Lens , A. Díaz , R. Vilana , A. Darnell , M. Varela , B. Sangro , J. L. Calleja , X. Forns , J. Bruix , J. Hepatol. 2016, 65, 719. 27084592 10.1016/j.jhep.2016.04.008

[5] A. J. Breugom , M. Swets , J. F. Bosset , L. Collette , A. Sainato , L. Cionini , R. Glynne‐Jones , N. Counsell , E. Bastiaannet , C. B. Broek , G. J. Liefers , H. Putter , C. J. Velde , Lancet Oncol. 2015, 16, 200. 25589192 10.1016/S1470-2045(14)71199-4

[6] R. P. Merkow , K. Y. Bilimoria , J. S. Tomlinson , J. L. Paruch , J. B. Fleming , M. S. Talamonti , C. Y. Ko , D. J. Bentrem , Ann. Surg. 2014, 260, 372. 24374509 10.1097/SLA.0000000000000378

[7] Y. W. Chen , Y. L. Su , S. H. Hu , S. Y. Chen , Adv. Drug Delivery Rev. 2016, 105, 190. 10.1016/j.addr.2016.05.022 27262922

[8] Q. Tian , M. Tang , Y. Sun , R. Zou , Z. Chen , M. Zhu , S. Yang , J. Wang , J. Wang , J. Hu , Adv. Mater. 2011, 23, 3542. 21735487 10.1002/adma.201101295

[9] K. Yang , L. Hu , X. Ma , S. Ye , L. Cheng , X. Shi , C. Li , Y. Li , Z. Liu , Adv. Mater. 2012, 24, 1868. 22378564 10.1002/adma.201104964

[10] C. Zhu , Y. Yang , M. Luo , C. Yang , J. Wu , L. Chen , G. Liu , T. Wen , J. Zhu , H. Xia , Angew. Chem. 2015, 127, 6279. 10.1002/anie.201501349 25824395

[11] Y. Sang , Z. Zhao , M. Zhao , P. Hao , Y. Leng , H. Liu , Adv. Mater. 2015, 27, 363. 25413166 10.1002/adma.201403264

[12] L. R. Hirsch , R. J. Stafford , J. A. Bankson , S. R. Sershen , B. Rivera , R. E. Price , J. D. Hazle , N. J. Halas , J. L. West , Proc. Natl. Acad. Sci. USA 2003, 100, 13549. 14597719 10.1073/pnas.2232479100 PMC263851

[13] N. W. S. Kam , M. O'Connell , J. A. Wisdom , H. Dai , Proc. Natl. Acad. Sci. USA 2005, 102, 11600. 16087878 10.1073/pnas.0502680102 PMC1187972

[14] T. S. Hauck , T. L. Jennings , T. Yatsenko , J. C. Kumaradas , W. C. W. Chan , Adv. Mater. 2008, 20, 3832.

[15] K. Dong , Z. Liu , Z. Li , J. Ren , X. Qu , Adv. Mater. 2013, 25, 4452. 23798450 10.1002/adma.201301232

[16] W. Yin , L. Yan , J. Yu , G. Tian , L. Zhou , X. Zheng , X. Zhang , Y. Yong , J. Li , Z. Gu , Y. Zhao , ACS Nano 2014, 8, 6922. 24905027 10.1021/nn501647j

[17] B. Li , K. Ye , Y. Zhang , J. Qin , R. Zou , K. Xu , X. Huang , Z. Xiao , W. Zhang , X. Lu , J. Hu , Adv. Mater. 2015, 27, 1339. 25639509 10.1002/adma.201404257

[18] G. Song , J. Hao , C. Liang , T. Liu , M. Gao , L. Cheng , J. Hu , Z. Liu , Angew. Chem., Int. Ed. 2016, 55, 2122. 10.1002/anie.201510597 26710169

[19] Q. Zou , M. Abbas , L. Zhao , S. Li , G. Shen , X. Yan , J. Am. Chem. Soc. 2017, 139, 1921. 28103663 10.1021/jacs.6b11382

[20] Q. Jia , J. Ge , W. Liu , X. Zheng , M. Wang , H. Zhang , P. Wang , ACS Appl. Mater. Interfaces 2017, 9, 21124. 28590721 10.1021/acsami.7b04360

[21] L. Wen , L. Chen , S. Zheng , J. Zeng , G. Duan , Y. Wang , G. Wang , Z. Chai , Z. Li , M. Gao , Adv. Mater. 2016, 28, 5072. 27136070 10.1002/adma.201506428

[22] S. Zhang , C. Sun , J. Zeng , Q. Sun , G. Wang , Y. Wang , Y. Wu , S. Dou , M. Gao , Z. Li , Adv. Mater. 2016, 28, 8927. 27560922 10.1002/adma.201602193

[23] F. Mao , L. Wen , C. Sun , S. Zhang , G. Wang , J. Zeng , Y. Wang , J. Ma , M. Gao , Z. Li , ACS Nano 2016, 10, 11145. 28024338 10.1021/acsnano.6b06067

[24] X. Jiang , S. Zhang , F. Ren , L. Chen , J. Zeng , M. Zhu , Z. Cheng , M. Gao , Z. Li , ACS Nano 2017, 11, 5633. 28525715 10.1021/acsnano.7b01032

[25] K. Yang , S. Zhang , G. Zhang , X. Sun , S. T. Lee , Z. Liu , Nano Lett. 2010, 10, 3318. 20684528 10.1021/nl100996u

[26] T. Zheng , G. G. Li , F. Zhou , R. Wu , J. J. Zhu , H. Wang , Adv. Mater. 2016, 28, 8218. 27459898 10.1002/adma.201602486

[27] X. Zhu , W. Feng , J. Chang , Y. W. Tan , J. Li , M. Chen , Y. Sun , F. Li , Nat. Commun. 2016, 7, 10437. 26842674 10.1038/ncomms10437 PMC4742858

[28] Q. Chen , L. Xu , C. Liang , C. Wang , R. Peng , Z. Liu , Nat. Commun. 2016, 7, 13193. 27767031 10.1038/ncomms13193 PMC5078754

[29] X. Zhao , C. X. Yang , L. G. Chen , X. P. Yan , Nat. Commun. 2017, 8, 14998. 28524865 10.1038/ncomms14998 PMC5454460

[30] L. Guo , I. Panderi , D. D. Yan , K. Szulak , Y. Li , Y. T. Chen , H. Ma , D. B. Niesen , N. Seeram , A. Ahmed , B. Yan , D. Pantazatos , W. Lu , ACS Nano 2013, 7, 8780. 24053214 10.1021/nn403202w PMC3870179

[31] L. Cheng , C. Wang , L. Feng , K. Yang , Z. Liu , Chem. Rev. 2014, 114, 10869. 25260098 10.1021/cr400532z

[32] Q. Chen , C. Wang , L. Cheng , W. He , Z. Cheng , Z. Liu , Biomaterials 2014, 35, 2915. 24412081 10.1016/j.biomaterials.2013.12.046

[33] H. O. H. Churchill , P. Jarillo‐Herrero , Nat. Nanotechnol. 2014, 9, 330. 24801536 10.1038/nnano.2014.85

[34] L. Li , Y. Yu , G. J. Ye , Q. Ge , X. Ou , H. Wu , D. Feng , X. H. Chen , Y. Zhang , Nat. Nanotechnol. 2014, 9, 372. 24584274 10.1038/nnano.2014.35

[35] J. Qiao , X. Kong , Z. X. Hu , F. Yang , W. Ji , Nat. Commun. 2014, 9, 4475. 10.1038/ncomms5475 PMC4109013 25042376

[36] D. Xiang , C. Han , J. Wu , S. Zhong , Y. Liu , J. Lin , X. A. Zhang , W. P. Hu , B. Özyilmaz , A. H. C. Neto , A. T. S. Wee , W. Chen , Nat. Commun. 2015, 6, 6485. 25761440 10.1038/ncomms7485

[37] X. Wang , A. M. Jones , K. L. Seyler , V. Tran , Y. Jia , H. Zhao , H. Wang , L. Yang , X. Xu , F. Xia , Nat. Nanotechnol. 2015, 10, 517. 25915195 10.1038/nnano.2015.71

[38] R. A. Doganov , E. C. O'farrell , S. P. Koenig , Y. Yeo , A. Ziletti , A. Carvalho , D. K. Campbell , D. F. Coker , K. Watanabe , T. Taniguchi , A. H. C. Neto , B. Özyilmaz , Nat. Commun. 2015, 6, 6647. 25858614 10.1038/ncomms7647

[39] Z. Sun , H. Xie , S. Tang , X. F. Yu , Z. Guo , J. Shao , H. Zhang , H. Huang , H. Wang , P. K. Chu , Angew. Chem., Int. Ed. 2015, 54, 11688. 10.1002/anie.201506154 26296530

[40] C. Sun , L. Wen , J. Zeng , Y. Wang , Q. Sun , L. Deng , C. Zhao , Z. Li , Biomaterials 2016, 91, 81. 27017578 10.1016/j.biomaterials.2016.03.022

[41] S. Comber , M. Gardner , K. Georges , D. Blackwood , D. Gilmour , Environ. Technol. 2013, 34, 1349. 24191467 10.1080/09593330.2012.747003

[42] D. L. Childers , J. Corman , M. Edwards , J. J. Elser , BioScience 2011, 61, 117.

[43] I. Pravst , Food Policy 2011, 36, 726.

[44] A. Castellanos‐Gomez , L. Vicarelli , E. Prada , J. O. Island , K. L. Narasimha‐Acharya , S. I. Blanter , D. J. Groenendijk , M. Buscema , G. A. Steele , J. V. Alvarez , H. W. Zandbergen , J. J. Palacios , H. S. van der Zant , 2D Mater. 2014, 1, 025001.

[45] J. O. Island , G. A. Steele , H. S. van der Zant , A. Castellanos‐Gomez , 2D Mater. 2015, 2, 011002.

[46] X. Ling , H. Wang , S. Huang , F. Xia , M. S. Dresselhaus , Proc. Natl. Acad. Sci. USA 2015, 112, 4523. 25820173 10.1073/pnas.1416581112 PMC4403146

[47] K. Shi , Y. L. Wang , Y. Qu , J. F. Liao , B. Y. Chu , H. P. Zhang , F. Luo , Z. Y. Qian , Sci. Rep. 2016, 6, 19077. 26752008 10.1038/srep19077 PMC4707506

[48] E. Zant , D. W. Grijpma , Acta Biomater. 2016, 31, 80. 26687979 10.1016/j.actbio.2015.12.014

[49] J. Shao , H. Xie , H. Huang , Z. Li , Z. Sun , Y. Xu , Q. Xiao , X. F. Yu , Y. Zhao , H. Zhang , H. Wang , P. K. Chu , Nat. Commun. 2016, 7, 12967. 27686999 10.1038/ncomms12967 PMC5056460

[50] Z. Guo , H. Zhang , S. Lu , Z. Wang , S. Tang , J. Shao , Z. Sun , H. Xie , H. Wang , X. F. Yu , P. K. Chu , Adv. Funct. Mater. 2015, 25, 6996.

[51] P. Yasaei , B. Kumar , T. Foroozan , C. Wang , M. Asadi , D. Tuschel , J. E. Indacochea , R. F. Klie , A. Salehi‐Khojin , Adv. Mater. 2015, 27, 1887. 25645510 10.1002/adma.201405150

[52] G. Hu , T. Albrow‐Owen , X. Jin , A. Ali , Y. Hu , R. C. Howe , K. Shehzad , Z. Yang , X. Zhu , R. I. Woodward , T. C. Wu , H. Jussila , J. B. Wu , P. Peng , P. H. Tan , Z. Sun , E. J. Kelleher , M. Zhang , Y. Xu , T. Hasan , Nat. Commun. 2017, 8, 278. 28819184 10.1038/s41467-017-00358-1 PMC5561124

[53] H. Xie , Z. Li , Z. Sun , J. Shao , X. F. Yu , Z. Guo , J. Wang , Q. Xiao , H. Wang , Q. Q. Wang , H. Zhang , P. K. Chu , Small 2016, 12, 4136. 27329254 10.1002/smll.201601050

[54] T. Liu , S. Shi , C. Liang , S. Shen , L. Cheng , C. Wang , X. Song , S. Goel , T. E. Barnhart , W. Cai , Z. Liu , ACS Nano 2015, 9, 950. 25562533 10.1021/nn506757x PMC4351725

[55] L. Chen , G. Zhou , Z. Liu , X. Ma , J. Chen , Z. Zhang , X. Ma , F. Li , H. M. Cheng , W. Ren , Adv. Mater. 2016, 28, 510. 26584241 10.1002/adma.201503678

[56] X. Yang , M. Yang , B. Pang , M. Vara , Y. Xia , Chem. Rev. 2015, 115, 10410. 26293344 10.1021/acs.chemrev.5b00193

[57] J. T. Robinson , S. M. Tabakman , Y. Liang , H. Wang , H. Sanchez Casalongue , D. Vinh , H. Dai , J. Am. Chem. Soc. 2011, 133, 6825. 21476500 10.1021/ja2010175

[58] L. Cheng , J. Liu , X. Gu , H. Gong , X. Shi , T. Liu , C. Wang , X. Wang , G. Liu , H. Xing , W. Bu , B. Sun , Z. Liu , Adv. Mater. 2014, 26, 1886. 24375758 10.1002/adma.201304497

[59] Z. Sun , Y. Zhao , Z. Li , H. Cui , Y. Zhou , W. Li , W. Tao , H. Zhang , H. Wang , P. K. Chu , X. F. Yu , Small 2017, 13, 1602896. 10.1002/smll.201602896 28060458

[60] Z. Zhang , J. Ni , L. Chen , L. Yu , J. Xu , J. Ding , Biomaterials 2011, 32, 4725. 21482434 10.1016/j.biomaterials.2011.03.046

[61] H. J. Moon , D. Y. Ko , M. H. Park , M. K. Joo , B. Jeong , Chem. Soc. Rev. 2012, 41, 4860. 22688789 10.1039/c2cs35078e

[62] D. Y. Ko , U. P. Shinde , B. Yeon , B. Jeong , Prog. Polym. Sci. 2013, 38, 672.

[63] Y. Yi , X. F. Yu , W. Zhou , J. Wang , P. K. Chu , Mater. Sci. Eng. R 2017, 120, 1.

[64] Z. Li , H. Huang , S. Tang , Y. Li , X. F. Yu , H. Wang , P. Li , Z. Sun , H. Zhang , C. Liu , P. K. Chu , Biomaterials 2016, 74, 144. 26454052 10.1016/j.biomaterials.2015.09.038

[65] Z. Fan , B. Liu , J. Wang , S. Zhang , Q. Lin , P. Gong , L. Ma , S. Yang , Adv. Funct. Mater. 2014, 24, 3933.

[66] M. C. Wu , A. R. Deokar , J. H. Liao , P. Y. Shih , Y. C. Ling , ACS Nano 2013, 7, 1281. 23363079 10.1021/nn304782d

[67] C. W. Hsiao , H. L. Chen , Z. X. Liao , R. Sureshbabu , H. C. Hsiao , S. J. Lin , Y. Chang , H. W. Sung , Adv. Funct. Mater. 2015, 25, 721.

[68] W. Yin , J. Yu , F. Lv , L. Yan , L. R. Zheng , Z. Gu , Y. Zhao , ACS Nano 2016, 10, 11000. 28024334 10.1021/acsnano.6b05810

