# Supplementary material for: Black‐Phosphorus‐Incorporated Hydrogel as a Sprayable and Biodegradable Photothermal Platform for Postsurgical Treatment of Cancer
Source: Adv Sci (Weinh). 2018 Mar 3;5(5):1700848. doi: 10.1002/advs.201700848 (PMC5978961; doi:10.1002/advs.201700848)
Supplement: Supplementary file 1 — Supplementary [file ADVS-5-1700848-s001.pdf]

Copyright WILEY-VCH Verlag GmbH & Co. KGaA, 69469 Weinheim, Germany, 2018.

Supporting Information

for *Adv. Sci.,* DOI: 10.1002/advs.201700848

Black-Phosphorus-Incorporated Hydrogel as a Sprayable and Biodegradable Photothermal Platform for Postsurgical Treatment of Cancer

Jundong Shao, Changshun Ruan, Hanhan Xie, Zhibin Li, Huaiyu Wang, Paul K. Chu, and Xue-Feng Yu**

Supporting Information

Black-Phosphorus-Incorporated Hydrogel as a Sprayable and Biodegradable Photothermal Platform for Post-Surgical Treatment of Cancer

Jundong Shao, Changshun Ruan, Hanhan Xie, Zhibin Li, Huaiyu Wang, Paul K. Chu and Xue-Feng Yu**

J. Shao, C. Ruan, H. Xie, Z. Li, Prof. H. Wang, Prof. X. -F. Yu,
Institute of Biomedicine and Biotechnology, Shenzhen Institutes of Advanced Technology, Chinese Academy of Sciences, Shenzhen, 518055, P.R. China.
E-mail: xf.yu@sitat.ac.cn (X. -F. Yu); hy.wang1@siat.ac.cn (H. Y. Wang)
Z. Li, Prof. P. K. Chu
Department of Physics and Department of Materials Science & Engineering, City University of Hong Kong, Tat Chee Avenue, Kowloon, Hong Kong, China.

Keywords: black phosphorus; two-dimensional materials; photothermal cancer therapy; near-infrared laser; thermo-sensitive hydrogel

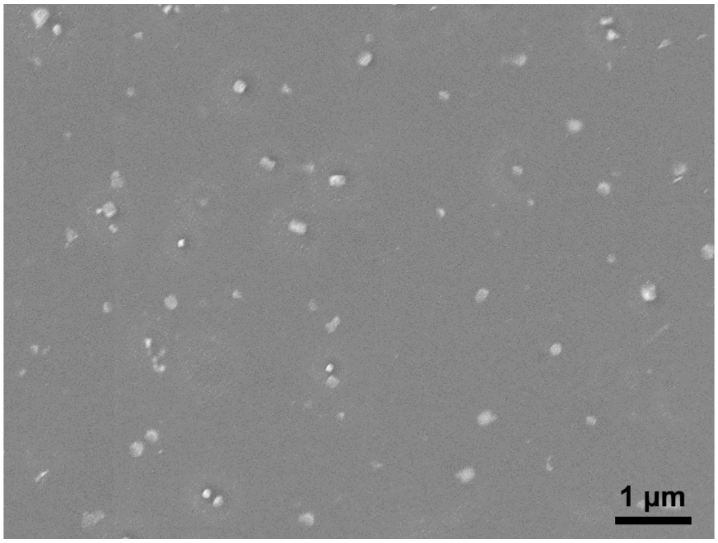

Figure S1. SEM image of the BP@PLEL hydrogel.

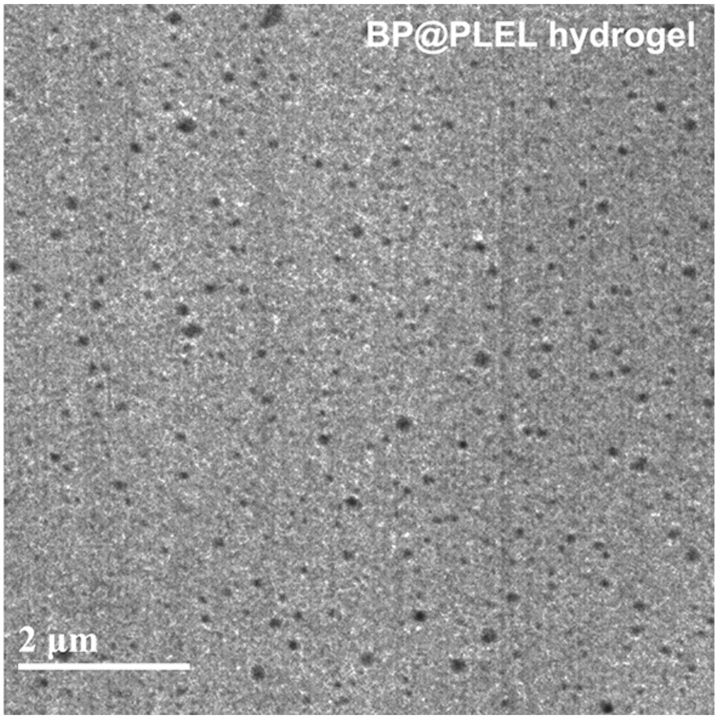

Figure S2. TEM image of the BP@PLEL hydrogel.

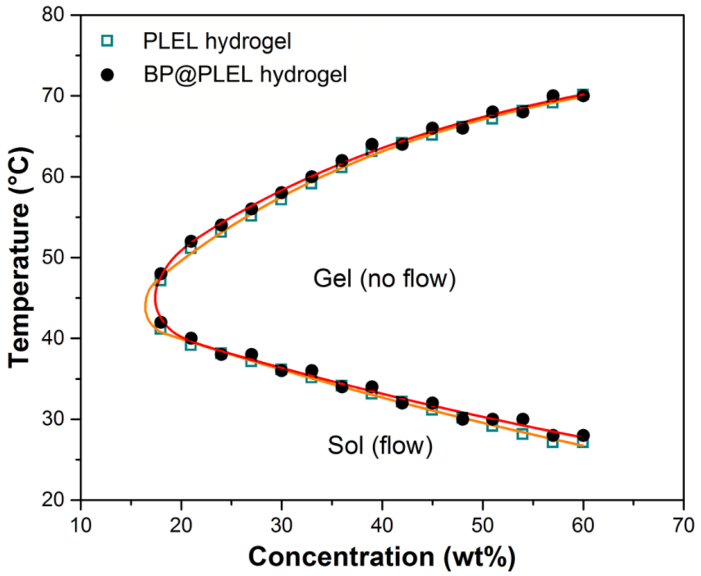

Figure S3. Sol-gel phase transition diagrams of the pure PLEL hydrogel and BP@PLEL hydrogel.

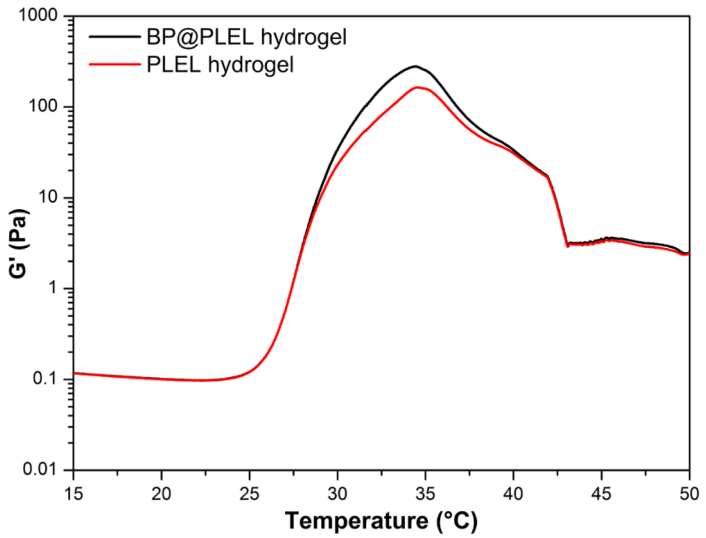

Figure S4. Temperature-dependent storage modulus (G') of the pure PLEL hydrogel and BP@PLEL hydrogel as a function of temperature.
